# Nano-Molybdenum Disulfide Enhances Antioxidant Defense and Aroma Formation in Fragrant Rice Under Cadmium Stress via Modulation of 2-Acetyl-1-Pyrroline Biosynthesis

**DOI:** 10.3390/antiox15070817

**Published:** 2026-06-29

**Authors:** Muhammad Imran, Muhammad Shoaib Rana, Xiangru Tang

**Affiliations:** 1 School of Medical Sciences, Shandong Xiehe University, Jinan 250109, China; 2 State Key Laboratory for Conservation and Utilization of Subtropical Agro-Bioresources, College of Agriculture, South China Agricultural University, Guangzhou 510642, China; 3 Department of Ecology, College of Natural Resources and Environment, South China Agricultural University, Guangzhou 510642, China

**Keywords:** nano-molybdenum disulfide, antioxidant defense, oxidative stress, 2-acetyl-1-pyrroline biosynthesis, fragrant rice, abiotic stress tolerance

## Abstract

2-acetyl-1-pyrroline (2-AP), the key volatile compound responsible for aroma in aromatic rice, is highly susceptible to abiotic stresses such as cadmium (Cd) toxicity. However, the potential role of molybdenum disulfide nanoflakes (MoS_2_FL) in regulating antioxidant defense and 2-AP biosynthesis under Cd stress remains largely unexplored. In this study, a pot experiment was conducted to evaluate the effects of foliar MoS_2_FL application on antioxidant defense, aroma formation, and Cd-stress mitigation in two fragrant rice cultivars, Meixiangzhan-2 and Basmati, grown in Cd-contaminated soil (50 mg kg^−1^). Cadmium stress significantly reduced key enzymes and precursors involved during 2-AP biosynthesis, including Δ1-pyrroline-5-carboxylate synthetase (P5CS), Δ1-pyrroline, pyrroline-5-carboxylic acid (P5C), diamine oxidase (DAO) and proline dehydrogenase (PRODH), along with downregulation of their associated genes. In contrast, foliar application of MoS_2_FL was associated with reduced Cd-induced oxidative stress, as indicated by increased antioxidant enzyme activities (SOD, POD, and CAT) and decreased malondialdehyde (MDA) accumulation. Moreover, MoS_2_FL increased precursor accumulation, enzymatic activities, and transcript abundance of genes associated with 2-AP biosynthesis, whereas gamma-aminobutyric acid (GABA) content, betaine aldehyde dehydrogenase (BADH) activity, and *BADH2* gene expression were significantly reduced. Consequently, MoS_2_FL application significantly increased 2-AP content by 44.47% in Meixiangzhan-2 and 39.94% in Basmati under Cd stress. These findings suggest that MoS_2_ nanoflakes may serve as a promising nano-enabled strategy to enhance antioxidant defense, improve aroma quality, and mitigate cadmium stress in fragrant rice, potentially through changes associated with the 2-AP biosynthesis pathway. This study highlights the potential application of nanomaterials in improving crop quality and stress resilience in sustainable agricultural systems.

## 1. Introduction

Aromatic rice is well-recognized for its distinctive taste, aroma, and consumer preference, which differentiate it from non-aromatic rice [1]. The aromatic characteristic of scented rice is described as popcorn-like and the major contributor to this unique aroma is considered to be 2-acetyl-1-pyrroline (2-AP). Some scientists believe that 2-AP content in aromatic rice defines its value and consumer demand in the global market [2,3]. Previous studies demonstrated that aromatic rice’s 2-AP content was highly sensitive to macro-micro nutrients, environmental factors, and cultural practices. For example, Mo et al. [4] reported that nitrogen application significantly affected 2-AP content, while water management practices have also been shown to play an important role in regulating 2-AP formation and accumulation [5,6]. Therefore, investigating the responses of the 2-AP biosynthesis pathway to stress conditions, particularly Cd toxicity, and its mitigation is important for maintaining aroma related quality traits in fragrant rice production.

**Figure 1:**
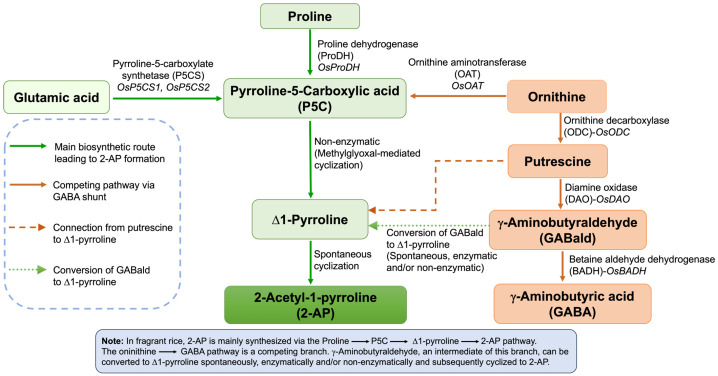
Schematic representation of 2-acetyl-1-pyrroline (2-AP) biosynthesis in fragrant rice.

The 2-AP synthesis in aromatic rice involves many physio-biochemical and molecular processes and the anticipated pathway has been shown in Figure 1. It has widely been accepted that aromatic rice’s 2-AP content is closely linked to the precursors, enzymatic activities and regulatory genes such as *BADH2*, *PRODH*, and *P5CS2*. Firstly, 2-AP precursors i.e., proline, glutamate, and ornithine are transformed to P5C by PRODH, P5CS, and OAT, and then methylglyoxal non-enzymatically converts P5C to Δ1-pyrroline and the end product as 2-acetyl-1-pyrroline [1]. Secondly, ODC catalyzes ornithine to Put, where DAO is the only enzyme that converts Put to gamma-aminobutyraldehyde (GABald), whose final fate is either Δ1-pyrroline or gamma-aminobutyric acid (GABA), depending on the BADH activity [7]. Some studies reported that 2-AP formation is highly associated with proline metabolism and positive correlation exists between proline (Pro) and 2-AP contents under different agronomic treatments such as water management, nitrogen fertilization, and zinc application [4,5]. On the other hand, some studies revealed that 2-AP enhancement was not always through proline but some other metabolic pathways such as Put and GABA metabolism [8].

The cornerstone of the best quality scented rice lies in enhancing the 2-AP levels and protecting the concerned enzymes and precursors in the 2-AP biosynthesis pathway. Currently, agriculture is undergoing a paradigm revolution from intensification to precision and decarbonization and the application of nanoparticles ranging in sizes from 1 to 1000 nm to agricultural plants are commonplace due to advancement in nanotechnology. These nanomaterials have the potential to increase crop output and growth while also improving crop protection effectiveness in adverse environments [9,10]. For instance, foliar application of Fe_2_O_3_ NPs increased soybean biological N fixation efficiency [11], CeO_2_ NPs protected plants from heat [12], salinity [13], and nitrogen extremities [14], and CuO nanosheets inhibited soybean sudden death syndrome [15]. Therefore, we anticipated that nanobiotechnology might be a helpful way to understand the advantages of nanomaterials on plant growth and the potential physiological and biochemical associations by which molybdenum disulfide nanoflakes (MoS_2_FL) may influence 2-AP production in fragrant rice under cadmium stress.

Molybdenum (Mo) is a vital element for higher plants, where it functions as active center for various nitrogen enzymes and has a great deal of promise for use in plants because of its minimal dosage requirements and substantial economic returns. Previous experiments on the Mo application have demonstrated its key importance as a stress resilient element to mitigate oxidative stresses, such as drought stress in wheat [16] and salinity stress in Chinese cabbage [17]. Similarly, MoS_2_ nanomaterials (the derivatives of Mo) are two-dimensional materials with unique physicochemical characteristics and exceptional ability due to their involvement in more than 50 different enzymes during various physio-biochemical and molecular processes [18,19,20,21]. However, the effects of MoS_2_FL on the sponsors of the 2-AP biosynthesis pathway in aromatic rice have still not been explored under Cd stress. Therefore, we hypothesized that the attributes of the 2-AP biosynthesis pathway are highly sensitive to Cd stress and that the application of MoS_2_FL may help maintain or improve the quality of fragrant rice in heavy metal-polluted soils. Based on this hypothesis, the present study aimed to evaluate the effects of MoS_2_FL on 2-AP biosynthesis, antioxidant defense systems, and the associated physiological, biochemical, and molecular responses of fragrant rice under Cd stress.

## 2. Material and Method

### 2.1. Experimental Site, Plant Husbandry, and Growth

**Figure 2:**
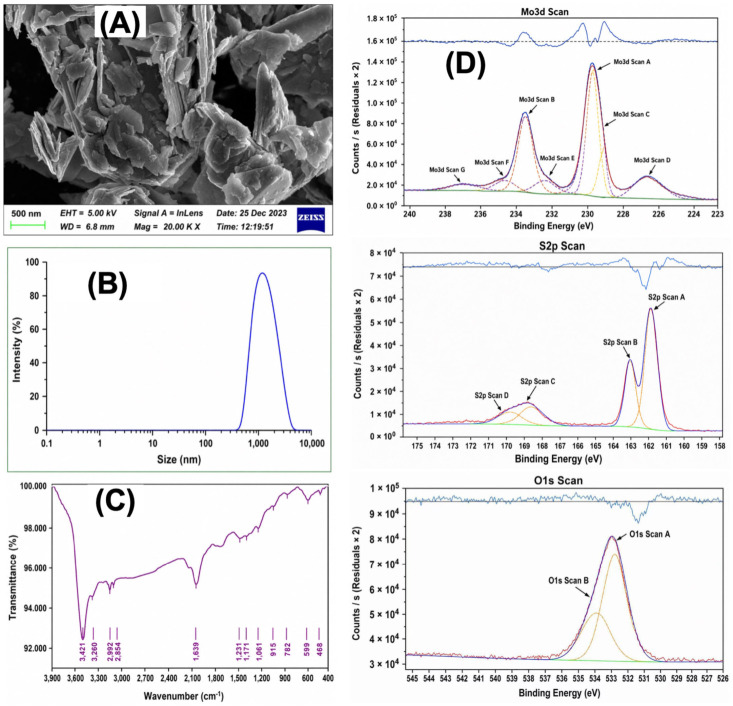
Characterization of molybdenum disulfide nanoflakes (MoS_2_FL). (**A**) Scanning electron microscopy (SEM) image showing the sheet-like morphology of MoS_2_FL. (**B**) Particle size distribution of MoS_2_FL determined by dynamic light scattering (DLS). (**C**) Fourier transform infrared (FTIR) spectrum of MoS_2_FL. (**D**) High-resolution X-ray photoelectron spectroscopy (XPS) spectra of Mo 3d, S 2p, and O 1s, confirming the chemical composition and elemental states of MoS_2_FL. In the XPS spectrum, the blue solid line represents the experimental spectrum, the red solid line represents the fitted spectrum, the green solid line represents the baseline, the dashed colored lines represent the deconvoluted component peaks, and the upper blue trace represents the residuals (×2).

The seeds and cultural materials for the two fragrant rice cultivars, Meixiagzhen-2 and Basmati, were taken from the College of Agriculture, SCAU. The experimental soil was collected from paddy fields with a long history of cultivation. The surface soil layer (0–20 cm) was collected, air-dried, thoroughly mixed, and used to fill plastic pots (25 cm height × 32 cm diameter), with 10 kg soil per pot. The soil contained 18.55 g kg^−1^ total potassium, 0.98 g kg^−1^ total phosphorus, 1.34 g kg^−1^ total nitrogen, 23.05 g kg^−1^ organic matter, 113.04 mg kg^−1^ available potassium, 27.36 mg kg^−1^ available phosphorus, and 92.63 mg kg^−1^ available nitrogen, and had a pH of 6.28 and a cadmium concentration of 0.0 mg kg^−1^. After 25 days of nursery growth, healthy and uniform-sized seedlings were transplanted into the pots. Five hills were established in each pot following a cross-shaped (+) planting pattern, with four hills positioned equidistantly around the pot and one hill in the center. Each hill consisted of 2–3 seedlings and was grown under the respective treatment conditions. The plant husbandry was done in the Experimental Research Farm of the College of Agriculture (114.02° E, 22.98° N). During the growing season, the average temperature ranged between 24 and 31 °C and the relative humidity was 75–89%. The molybdenum disulfide nanoflakes (MoS_2_FL) with particle sizes of <1 µm were bought from Nanjing XFNANO Materials Tech Co., Ltd. (Nanjing, Jiangsu, China). (Figure 2). The experimental treatments were comprised of foliar sprays of MoS_2_FL (0 and 1 µM) and basal doses of Cd (0 and 50 mg kg^−1^). The experimental soil was thoroughly mixed with Cd (50 mg kg^−1^ of soil) in the form of CdCl_2_·2.5H_2_O thirty days prior to the transplanting of rice seedlings. The Cd level used in the present study was selected based on our previous fragrant rice pot experiment under Cd stress, in which a higher Cd concentration of 100 mg kg^−1^ soil was used to evaluate molybdenum-mediated mitigation of Cd toxicity, 2-AP formation, grain quality, and yield-related traits [22]. Therefore, a lower Cd level of 50 mg kg^−1^ soil was adopted in the present study to establish a clear but physiologically manageable Cd stress condition suitable for biochemical, molecular, transcriptomic, and metabolomic analyses under foliar MoS_2_FL application.

To establish anaerobic and puddled soil conditions, a 2–3 cm water layer was maintained in all experimental pots throughout the growing period. Foliar applications of MoS_2_FL were initiated 10 days after transplanting and applied to both the adaxial and abaxial leaf surfaces using a hand sprayer. A total of six spray applications were performed at 15-day intervals. The MoS_2_FL suspension was sprayed uniformly until the leaves were thoroughly wetted. At maturity, fresh panicles were harvested and immediately frozen and stored at −80 °C for subsequent biochemical and molecular analyses.

### 2.2. Preparation and Characterization of MoS_2_FL

The physicochemical properties of molybdenum disulfide nanoflakes (MoS_2_FL) were characterized by Guangzhou Puchuan Testing Technology Co., Ltd. (Guangzhou, China). Surface morphology was examined using a field-emission scanning electron microscope (FE-SEM; Sigma 300, Carl Zeiss, Oberkochen, Germany). Prior to analysis, MoS_2_FL samples were dispersed in ethanol, deposited onto clean silicon wafers, air-dried, mounted on aluminum stubs using conductive adhesive, and sputter-coated with platinum to improve conductivity before observation.

The hydrodynamic particle size distribution of MoS_2_FL suspensions was determined using dynamic light scattering (DLS) with a Malvern Zetasizer Nano particle size analyzer (Malvern Instruments, Malvern, UK). Particle size measurements were performed according to the dynamic light scattering principle using approximately 1 mL of sample suspension.

Fourier-transform infrared spectroscopy (FTIR) was conducted using a Bruker Tensor 27 spectrometer (Bruker, Optik, GmbH, Ettlingen, Germany). Samples were prepared using the KBr pellet method at a sample-to-KBr ratio of 1:50. Spectra were recorded over the range of 400–4000 cm^−1^ with a spectral resolution of 0.25 cm^−1^ and 16 scans.

The elemental composition and surface chemical states of MoS_2_FL were analyzed using X-ray photoelectron spectroscopy (XPS) with a Thermo Scientific K-Alpha spectrometer (Thermo Fisher Scientific, Waltham, MA, USA) equipped with an Al Kα radiation source (1486.8 eV). Analyses were conducted at an operating voltage of 15 kV and a current of 10 mA under ultra-high vacuum conditions (~2 × 10^−9^ mbar). Survey and high-resolution spectra were collected and processed using Avantage software version 5.9925 (Thermo Fisher Scientific).

### 2.3. Quantification of 2-AP Content

Dichloromethane and sodium sulphate were used to normalize fragrant rice grains for 2-AP measurement. The SDE technique was used to determine the 2-AP concentration using a GCMS-QP2010 Plus Gas Chromatograph Mass Spectrometer (Shimadzu Corporation, Kyoto, Japan) [22].

### 2.4. Determination of Proline, Δ1-Pyrroline, P5C, and GABA Contents

The Δ1-pyrroline content was determined according to [5]. In short, the plant samples were homogenized with PBS and γ-aminobenzaldehyde (mixed with 0.02 mM phosphate buffer). The reaction solution was kept for 30 min at 25 °C and the absorbance was measured at 430 nm in the spectrophotometer. Proline was extracted in fresh plant samples by homogenizing with ninhydrin and sulfosalicylic acid. The absorbance was read at 520 nm to calculate the proline levels [23]. To determine the P5C contents, we followed the method of [24]. Shortly, the plant samples were extracted with β-mercaptoethanol (1%), Tris-HCl (50 mM), tritonX100 (1%), and glycerol (10%). The extracted solution was reacted with γ-aminobenzaldehyde (40 mM) and TCA (10%). The P5C content was measured using the extinction coefficient 2.58 mM^−1^ cm^−1^. The GABA content was measured by the method of [25]. The plant samples were extracted with KOH (1M), lanthanum chloride (60 mM), and ethanol (60%). The extracted solution was treated with phenol (6%), sodium hypochloride (available chlorine, 10%) and borate buffer (0.2 M, pH 10.0). The GABA contents were measured after reading the absorbance at 645 nm.

### 2.5. Determination of PRODH, BADH, DAO, OAT and P5CS Enzymes

The activities of PRODH and BADH enzymes were determined using commercial enzyme activity assay kits (ProDH Activity Assay Kit, R45403-96S; BADH Activity Assay Kit, R45169-48S; Shanghai Yuanye Bio-Technology Co., Ltd., Shanghai, China) according to the manufacturers’ instructions. Absorbance values were recorded using a BioTek EPOCH microplate spectrophotometer (BioTek Instruments, Inc., Winooski, VT, USA) at 340 and 450 nm for PRODH and BADH, respectively. For the determination of DAO, OAT, and P5CS activities, fresh plant samples were homogenized in 50 mM Tris–HCl buffer containing 5% PVP, 3.0 mM EDTA-Na_2_, 1% glycerol, 1.0 mM DTT, 1.0 mM KCl and 7.0 mM MgCl_2_. The homogenate was centrifuged and the resulting supernatant was used for enzyme assays. The activities of P5CS, OAT, and DAO were measured according to [5,26,27] and reading the absorbances at 340, 440, and 550 nm respectively.

### 2.6. Real-Time Quantitative RT-PCR

Fresh aromatic rice plants were sampled for total RNA extraction using RNAiso Plus reagent (Takara Bio Inc., Kusatsu, Shiga, Japan). RNA quality assessment, cDNA synthesis, and quantitative real-time PCR (qRT-PCR) analyses were performed according to previously published protocol [22]. Primer sequences of the target genes and the internal reference gene ACTIN are provided in Table 1. Three independent biological replicates were analyzed for each treatment. Relative gene expression levels were normalized against ACTIN and calculated using the 2^−ΔΔCt^ method.

### 2.7. Quantification of Enzymatic Antioxidants and Lipid Peroxidation

To measure the enzymatic antioxidant, fresh plant samples were homogenized with a mortar and pestle in cold phosphate buffered saline. The supernatant was separated from crude fiber and the aliquot was used to quantify superoxide dismutase, catalase, and peroxidase by following the previous methods [28]. The malondialdehyde (MDA) levels were estimated according to earlier described method [29].

### 2.8. Determination of Cd Concentration in Plant Tissues and Grains

To determine Cd concentration, roots, shoots, leaves, and grains of fragrant rice plants were collected at maturity, oven-dried to constant weight, and ground into fine powder. Approximately 0.2 g of dried sample was digested in a microwave digestion system (MLS 1200, Milestone, FKV, Varese, Italy) using a di-acid mixture of HNO_3_:HClO_4_ (5:1, *v*/*v*). Cadmium concentrations in the digested samples were determined by inductively coupled plasma mass spectrometry (ICP-MS; ELAN DRC-e, Perkin-Elmer Sciex, Wilmington, DE, USA) and expressed as μg g^−1^ dry weight (DW).

### 2.9. Statistical Analysis

All data were expressed as the mean ± standard error (SE) of four biological replicates. Statistical analyses were performed using Statistix 8.1 (Analytical Software, Tallahassee, FL, USA). The effects of cultivar, Cd treatment, MoS_2_FL treatment, and their interactions were evaluated using three-way analysis of variance (ANOVA). When significant differences were detected, mean comparisons were performed using Tukey’s honestly significant difference (HSD) test at the 5% probability level (*p* < 0.05). Different letters above the bars indicate significant differences among treatments according to Tukey’s HSD test at *p* < 0.05. Graphs were generated and Pearson’s product-moment correlation analyses were performed using SigmaPlot version 16.0 (Systat Software Inc., San Jose, CA, USA). Correlation coefficients (r) and corresponding *p*-values were calculated using all observations from both cultivars and treatments (n = 32). Statistical significance was determined at *p* < 0.05.

## 3. Results

### 3.1. Tissue-Specific Accumulation of 2-AP in Fragrant Rice Cultivars

**Figure 3:**
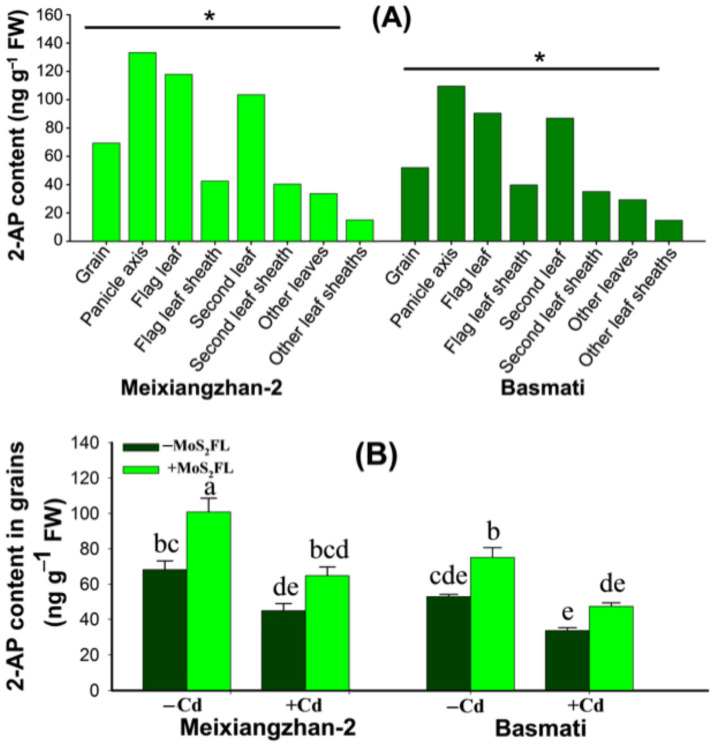
The 2-acetyl-1-pyrroline (2-AP) content in different plant tissues of fragrant rice cultivars Meixiangzhan-2 and Basmati (**A**), and the effects of foliar application of molybdenum disulfide nanoflakes (MoS_2_FL) on grain 2-AP content under cadmium (Cd) stress (50 mg kg^−1^ soil) (**B**). Values represent means ± SE of four biological replicates (n = 4). For (**A**), Tukey’s honestly significant difference (HSD) test was used to compare 2-AP content between cultivars at *p* < 0.05; the asterisk (*) indicates a significant difference. For (**B**), different lowercase letters indicate significant differences among treatments according to Tukey’s HSD test at *p* < 0.05.

The 2-AP content in different plant tissues of fragrant rice is shown in Figure 3. To compare tissue-specific 2-AP accumulation, data from all treatments and biological replicates were pooled for each cultivar. The results showed clear cultivar-dependent differences in 2-AP accumulation, with Meixiangzhan-2 exhibiting significantly higher 2-AP content than Basmati across the examined plant tissues. This observation suggests that Meixiangzhen-2 may have a greater genetic or metabolic potential for 2-AP accumulation compared with Basmati under the tested conditions. However, the 2-AP content in various plant parts followed the same order in both fragrant rice cultivars as: panicle axis > flag leaf > second leaf > grain > flag leaf sheath > second leaf sheath > other leaves > other leaf sheaths (Figure 3A). The Cd toxicity significantly reduced the 2-AP concentrations in freshly harvested grains relative to without Cd stress; however, foliar sprays of MoS_2_FL significantly improved the 2-AP in both aromatic rice cultivars. Under Cd stress, foliar sprays of MoS_2_FL increased the 2-AP contents by 44.47% and 39.94% in Meixiangzhan-2 and Basmati rice cultivars, respectively, as compared to those without MoS_2_FL treatments (Figure 3B).

### 3.2. Effects of Foliar Application of MoS_2_FL on 2-AP-Related Precursors

**Figure 4:**
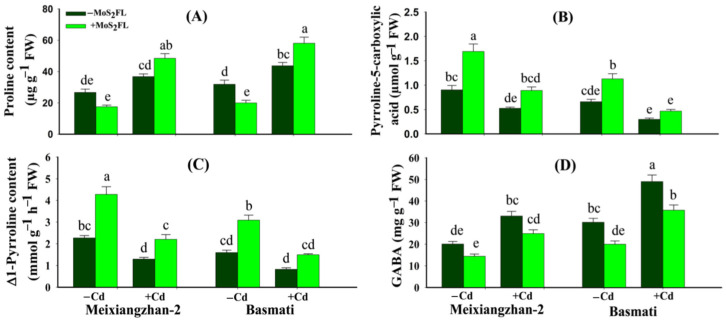
Effects of foliar application of molybdenum disulfide nanoflakes (MoS_2_FL) on proline content (**A**), pyrroline-5-carboxylic acid (P5C) content (**B**), Δ1-pyrroline content (**C**), and γ-aminobutyric acid (GABA) content (**D**) in fragrant rice cultivars Meixiangzhan-2 and Basmati under cadmium (Cd) stress (50 mg kg^−1^ soil). Values represent means ± SE of four biological replicates (n = 4). Different lowercase letters indicate significant differences among treatments according to Tukey’s HSD test at *p* < 0.05.

The 2-AP content in plant tissues depend upon the precursors; Δ1-pyrroline, P5C, proline, and GABA during the 2-AP biosynthesis pathway (Figure 4). In this study, Cd toxicity significantly increased proline and GABA contents (Figure 4A,D), whereas it decreased Δ1-pyrroline and P5C contents in both cultivars as compared to without Cd stress. Moreover, exogenous spraying of MoS_2_FL further increased proline contents under Cd stress while significant reductions were recorded under without Cd stressed aromatic rice plants of both cultivars (Figure 4A). In contrast to proline contents, exogenous application of MoS_2_FL significantly reduced GABA contents both under with or without Cd stress. The Basmati cultivar accumulated significantly higher proline and GABA contents concerning Meixiangzhen-2 fragrant rice cultivar under Cd stress, suggesting that Basmati cultivar is more susceptible to cadmium stress (Figure 4A,D). In contrast to proline and GABA contents, Cd stress significantly reduced Δ1-pyrroline and P5C contents in both cultivars compared to without Cd stress, while exogenous spraying of MoS_2_FL significantly (*p* < 0.05) increased their content irrespective of Cd stress. Under Cd toxicity, foliar sprays of MoS_2_FL increased proline, P5C and Δ1-pyrroline contents by 31.65%, 68.82%, and 70.09% in Meixiangzhan-2 and 32.74%, 55.38%, and 79.61% in Basmati respectively while reducing GABA content by 24.62% and 27.01% in Meixiangzhan-2 and Basmati respectively (Figure 4). Overall, Meixiangzhan-2 showed relatively higher accumulation of 2-AP-related metabolites under both Cd and non-Cd conditions than Basmati, suggesting that Meixiangzhan-2 may have a stronger capacity to accumulate 2-AP-related metabolites under the tested conditions.

### 3.3. Impacts of Foliar Sprays of MoS_2_FL on the Enzymatic Activity and the Transcript Levels of 2-AP Biosynthesis Enzymes Under Cd Stress

**Figure 5:**
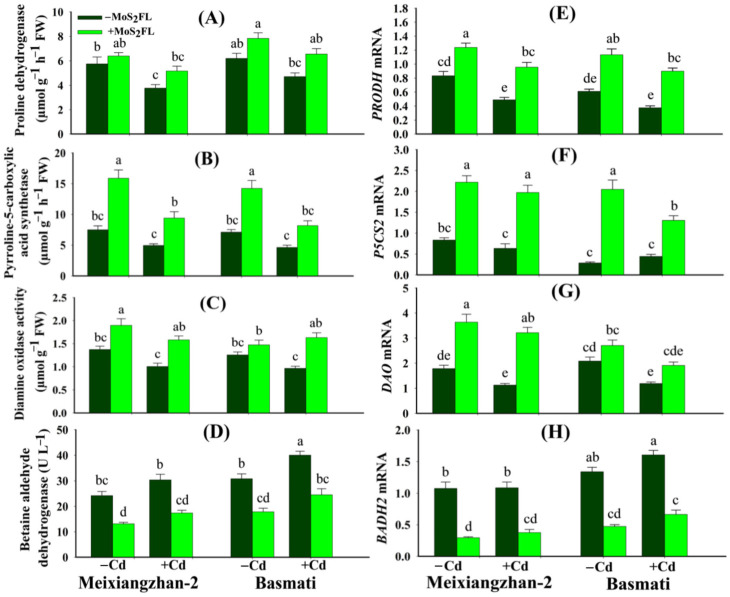
Effects of foliar application of molybdenum disulfide nanoflakes (MoS_2_FL) on the activities of 2-AP biosynthesis-related enzymes, including proline dehydrogenase (PRODH) (**A**), pyrroline-5-carboxylic acid synthetase (P5CS) (**B**), diamine oxidase (DAO) (**C**), and betaine aldehyde dehydrogenase (BADH) (**D**), as well as the relative transcript abundances of *PRODH* (**E**), *P5CS2* (**F**), *DAO* (**G**), and *BADH2* (**H**) in fragrant rice cultivars Meixiangzhan-2 and Basmati under cadmium (Cd) stress (50 mg kg^−1^ soil). Values represent means ± SE of four biological replicates (n = 4). Different lowercase letters indicate significant differences among treatments according to Tukey’s honestly significant difference (HSD) test at *p* < 0.05.

The results revealed that foliar sprays of MoS_2_FL under Cd toxicity showed differential effects on the 2-AP biosynthesis enzymes; BADH, DAO, PRODH, and P5CS (Figure 5). Relative to Cd treatments, Cd toxicity significantly decreased P5CS, DAO, and PRODH activity, but increased BADH activity. However, foliar sprays of MoS_2_FL significantly increased PRODH, P5CS, and DAO activity, while it reduced BADH activity in both cultivars, suggesting that exogenous sprays of MoS_2_FL mitigated the Cd-induced adversities on the 2-AP biosynthesis pathway. Under Cd toxicity, exogenous sprays of MoS_2_FL increased PRODH activity by 37.04% and 39.03%, P5CS activity by 89.49% and 75.97%, and DAO activity by 57.06% and 68.55%, while BADH activity decreased by 42.64% and 38.71% in Meixiangzhen-2 and Basmati rice cultivars, respectively (Figure 5A–D). Similarly, real-time PCR analysis showed that Cd stress reduced the relative transcript abundances of PRODH and DAO, whereas P5CS2 and BADH2 were less affected under Cd stress in both fragrant rice cultivars. Foliar application of MoS_2_FL increased the transcript abundances of *PRODH, P5CS2*, and *DAO*, while decreasing *BADH2* transcript abundance under both non-Cd and Cd-stress conditions (Figure 5E–H). These transcriptional changes were generally consistent with the observed changes in enzyme activities and 2-AP-related metabolites, suggesting that MoS_2_FL application was associated with coordinated changes in the 2-AP biosynthesis pathway.

### 3.4. Impacts of Foliar Sprays of MoS_2_FL on Antioxidant Defense of Fragrant Rice Cultivars Under Cd Toxicity

**Figure 6:**
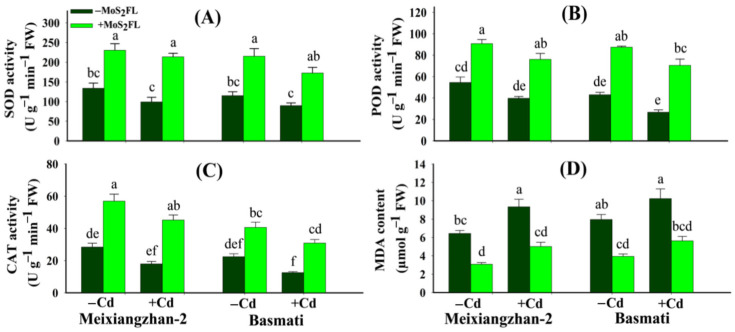
Effects of foliar application of molybdenum disulfide nanoflakes (MoS_2_FL) on the activities of superoxide dismutase (SOD) (**A**), peroxidase (POD) (**B**), catalase (CAT) (**C**), and malondialdehyde (MDA) content (**D**) in fragrant rice cultivars Meixiangzhan-2 and Basmati under cadmium (Cd) stress (50 mg kg^−1^ soil). Values represent means ± SE of four biological replicates (n = 4). Different lowercase letters indicate significant differences among treatments according to Tukey’s honestly significant difference (HSD) test at *p* < 0.05.

Because 2-AP biosynthesis is highly sensitive to stress conditions, the antioxidant defense responses of fragrant rice cultivars were evaluated under Cd stress and foliar MoS_2_FL application (Figure 6). Cd stress significantly reduced the activity of POD and CAT antioxidant enzymes while SOD activity was not significantly affected as compared to without Cd toxicity. However, foliar sprays of MoS_2_FL significantly increased enzymatic activity in both cultivars. Under Cd toxicity, MoS_2_FL application enhanced SOD activity by 115.10% and 91.67%, POD activity by 91.29% and 163.11%, and CAT activity by 150.75% and 144.67% in Meixiangzhen-2 and Basmati, respectively. Conversely, Cd stress significantly increased MDA content in both cultivars as compared to those without Cd treatments (Figure 6D). However, foliar sprays of MoS_2_FL reduced the MDA contents by 46.25% in Meixiangzhen-2 and 44.84% in Basmati under Cd toxicity. These results suggest that MoS_2_FL application was associated with strengthened antioxidant defense and reduced lipid peroxidation in fragrant rice under Cd toxicity.

To further examine whether foliar application of MoS_2_FL influenced Cd accumulation and translocation within the plant, Cd concentrations were determined in roots, shoots, leaves, and grains (Supplementary Figure S1). Under Cd stress, MoS_2_FL application did not significantly alter root Cd concentration, whereas it reduced Cd accumulation in shoots, leaves, and grains, with the most evident reductions observed in leaves and grains of both cultivars. These findings suggest that the ameliorative effects of MoS_2_FL under Cd stress were associated, at least in part, with a reduction in Cd accumulation in aerial tissues and grains.

### 3.5. Correlation Analysis

**Figure 7:**
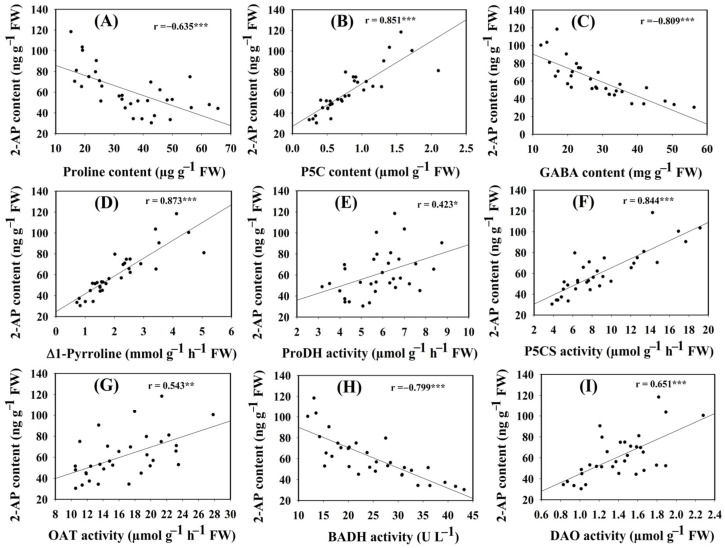
Pearson correlation analysis between 2-acetyl-1-pyrroline (2-AP) content and key precursors and enzymes involved in 2-AP biosynthesis in fragrant rice. (**A**) Proline, (**B**) pyrroline-5-carboxylic acid (P5C), (**C**) γ-aminobutyric acid (GABA), (**D**) Δ1-pyrroline, (**E**) proline dehydrogenase (PRODH), (**F**) pyrroline-5-carboxylate synthetase (P5CS), (**G**) ornithine aminotransferase (OAT), (**H**) betaine aldehyde dehydrogenase (BADH), and (**I**) diamine oxidase (DAO). Correlation analyses were performed using all observations from both cultivars and treatments (n = 32). Values represent Pearson correlation coefficients (r). Regression lines indicate linear relationships between variables. *, ** and *** indicate significance at *p* < 0.05, *p* < 0.01 and *p* < 0.001, respectively.

Pearson’s product–moment correlation analysis was performed to investigate the relationships between 2-acetyl-1-pyrroline (2-AP) content and key precursors and enzymes involved in the 2-AP biosynthetic pathway (Figure 7). The results showed that 2-AP content was significantly negatively correlated with proline (r = −0.635, *p* < 0.001), γ-aminobutyric acid (GABA) (r = −0.809, *p* < 0.001), and betaine aldehyde dehydrogenase (BADH) activity (r = −0.799, *p* < 0.001). In contrast, significant positive correlations were observed between 2-AP content and pyrroline-5-carboxylic acid (P5C) (r = 0.851, *p* < 0.001), Δ1-pyrroline (r = 0.873, *p* < 0.001), proline dehydrogenase (PRODH) activity (r = 0.423, *p* = 0.0159), pyrroline-5-carboxylate synthetase (P5CS) activity (r = 0.844, *p* < 0.001), ornithine aminotransferase (OAT) activity (r = 0.543, *p* = 0.0013), and diamine oxidase (DAO) activity (r = 0.651, *p* < 0.001). Among these parameters, Δ1-pyrroline, P5C, and P5CS activity exhibited the strongest positive associations with 2-AP accumulation, whereas GABA and BADH activity showed strong negative relationships. These findings indicate that enhanced 2-AP biosynthesis is closely associated with increased precursor availability and enzymatic activities involved in aroma formation, while GABA accumulation and BADH activity may negatively regulate 2-AP production in fragrant rice.

## 4. Discussion

A key aromatic compound responsible for the characteristic aroma of fragrant rice is 2-acetyl-1-pyrroline (2-AP). The biosynthesis and accumulation of 2-AP in fragrant rice cultivars are complex processes influenced by various agronomic practices and environmental factors [30,31]. Previous studies have shown that 2-AP accumulation is highly responsive to nitrogen application rates, water management regimes, alternate wetting and drying conditions, and shading periods [5,32,33]. More specifically, previous studies on 2-AP accumulation in fragrant rice have generally followed two major approaches. The first approach involves exposing rice plants to stress conditions such as lead stress, cadmium stress, salinity, or shading, whereas the second approach involves the application of nutritional elements such as nitrogen, silicon, manganese, molybdenum, and zinc. In this context, the present study evaluated the effects of foliar MoS_2_FL application on 2-AP accumulation, related precursor metabolites, and associated enzyme activities in fragrant rice under cadmium stress.

The present study revealed that the 2-AP concentrations were observed in all the plant parts of scented rice. These results coincide with earlier reports that 2-AP exists in all the aerial parts of aromatic rice plants except roots [4] and the factors affecting 2-AP content in aromatic rice plants include planting site, cultivar, genotype, and even the part of the plant [34,35]. Similarly, ref. [36] detected 2-AP in three distinct fragrant rice varieties’ leaves, stems, panicles, and grains. Furthermore, the results of current experiment revealed following order of 2-AP contents in various plant parts: panicle axis > flag leaf > second leaf > grain > flag leaf sheath > second leaf sheath > other leaves > other leaf sheaths. The highest 2-AP contents were accumulated in the panicle axis while the least were in other leaf sheaths (Figure 3), which agrees with Mo et al. [4] but contradicts the reports of [28]. Moreover, the reduced 2-AP concentration in Basmati compared to Meixiangzhen-2 might be attributed to genotypic difference, and agrees with the findings of [35].

The synthesis and accumulation of 2-AP in fragrant rice are closely associated with key precursors, including proline, Δ1-pyrroline, P5C, and GABA, which play important roles in the 2-AP biosynthesis pathway [28]. In the present study, Cd stress significantly reduced 2-AP content in both fragrant rice cultivars, whereas foliar application of MoS_2_FL was associated with reduced Cd toxicity and increased 2-AP content. The Cd-induced reduction in 2-AP content may be related to altered precursor availability, changes in the activities of 2-AP biosynthesis-related enzymes, and weakened antioxidant defense under stress conditions. These findings are consistent with previous studies reporting reduced 2-AP accumulation in aromatic rice under environmental stress conditions [5]. The MoS_2_ has been widely applied in industry and has great potential applications in environment due to their extraordinary properties [37]. Furthermore, molybdenum, an essential and anti-stress micronutrient, has attracted a lot of attention because of its major role in a variety of plant growth and production processes as well as its enhancement of oxidative stress resistance under heavy metals, drought, salinity and cold stresses [38]. Therefore, the improvement in 2-AP content under MoS_2_FL application may be associated with increased precursor levels and enhanced activities of enzymes related to the 2-AP biosynthesis pathway.

The supplementary analysis of Cd distribution provides further insight into the effect of MoS_2_FL on Cd partitioning within rice plants. Foliar application of MoS_2_FL had little effect on root Cd accumulation, suggesting limited influence on initial Cd uptake under foliar treatment. However, a clear reduction in Cd accumulation in aerial tissues and grains was observed, indicating that MoS_2_FL may primarily regulate Cd translocation rather than root uptake processes. This pattern suggests a potential role of MoS_2_FL in limiting the movement of Cd from roots to photosynthetically active tissues and edible organs. Moreover, cultivar-dependent differences were evident, as Meixiangzhan-2 showed greater Cd retention in roots but lower Cd accumulation in shoots, leaves, and grains compared with Basmati, implying differential Cd partitioning efficiency between cultivars under Cd stress.

Proline has been considered a crucial precursor of 2-AP, and its metabolism was believed to be closely associated to 2-AP synthesis in fragrant rice [5,34]. In this experiment, the Cd stress increased the proline contents and foliar sprays of MoS_2_FL further increased proline in both cultivars. However, in contrast to proline, Cd stress decreased 2-AP contents, and the possible reason might be due to significantly lower PRODH activity and resultantly reduced P5C contents, which is the immediate precursor during 2-AP synthesis (Figure 1). Moreover, the transcript level of PRODH, which is an important gene in proline metabolism [39], reduced under Cd stress, while foliar sprays of MoS_2_FL were associated with restored PRODH levels and higher 2-AP content. These findings agree with earlier studies, which demonstrated that the expression of PRODH was concomitantly related to 2-AP production in scented rice grains [5]. Furthermore, the increased 2-AP contents under MoS_2_FL treatments might be attributed to the increased contents of P5C and proline (Figure 4). P5C is a mediate in both proline biosynthesis and degradation. A previous study showed that P5C formed by proline dehydrogenase encoded by PRODH from the *Bacillus subtilis* ssp. natto expressed in *Escherichia coli* is a 2-AP precursor [40]. The present study showed that higher proline content under MoS_2_FL treatment was accompanied by induced P5C and Δ1-pyrroline accumulation, which may have contributed to higher 2-AP content in fragrant rice and similar findings were reported in other environmental conditions [5].

Li et al. [41] reported that higher 2-AP concentrations in fragrant rice plants might be associated with greater activity of enzymes involved in its biosynthesis, and found a positive correlation between 2-AP contents and OAT, P5CS, and PRODH activity. In addition, ref. [7] discovered that when compared to non-fragrant rice cultivars, fragrant rice cultivars had higher P5C levels as well as increased OAT, P5CS, and PRODH activity. Ref. [42] reported Δ1-pyrroline to be a limiting factor for 2-AP biosynthesis. The present study revealed that down-regulation of GABA and up-regulation of Δ1-pyrroline and P5C contents have resulted in increased 2-AP production. Furthermore, it has been reported that P5CS plays a key role in 2-AP accumulation and the transcriptional expressions of functional gene *P5CS2* are associated with 2-AP biosynthesis in aromatic rice plants [29]. According to our findings, *P5CS2* gene expressions were not significantly affected under Cd stress, whilst foliar sprays of MoS_2_FL significantly increased *P5CS2* expressions and showed positive correlations with 2-AP concentration. Therefore, we suggest that the increased 2-AP contents under foliar sprays of MoS_2_FL were mainly due to the improvement in *P5CS2* transcript levels. These results agree with the reports of [43], that the overexpression of *P5CS2* gene increased the amount of 2-AP in transgenic aromatic rice.

The DAO converts Put into GABald, which is spontaneously cyclized to either GABA or Δ1-pyrroline, according to the functionality of BADH enzyme [7]. The non-functional BADH enzyme encouraged the buildup of Δ1-pyrroline, which led to increased fragrance in scented rice varieties, while functional *BADH2* converted GABald into GABA to suppress aroma synthesis in non-fragrant cultivars [44]. The current study reveals that Cd stress increased GABA content and the reason might be due to higher BADH activity under Cd stress, and that it coincides with lower Δ1-pyrroline contents and resultingly lower 2-AP contents (Figure 3 and Figure 4). However, MoS_2_FL-induced improvement in 2-AP contents might be due to higher *BADH2* gene expression and the activity of BADH enzyme which might have switched Δ1-pyrroline synthesis rather than GABA production. Nevertheless, studies have reported controversial relationships between GABA and 2-AP in scented rice, and the possible reason is that the database of Kyoto Encyclopedia of Genes and Genomes (KEGG), (https://www.genome.jp/kegg/, accessed on 23 June 2026), revealed several pathways to form GABA in plants. Therefore, ref. [32] reported that shading treatments considerably improved the contents of GABA and 2-AP in fragrant rice and found positive correlations between them, indicating that the “GABA shunt” is the primary pathway for GABA production and/or that other enzymes may have catalyzed GABald conversion to GABA [45]. Ref. [28] reported that salt stress activated 2-AP but had no effect on GABA contents. In addition, low GABA contents were recorded in isogenic fragrant rice lines relative to non-fragrant lines, and there were negative correlations between the GABA and 2-AP concentrations [7]. Similarly, in this study, 2-AP concentrations increased with a decrease in GABA contents and there existed a negative correlation between GABA and 2-AP concentrations under foliar sprays of MoS_2_FL to Cd stressed fragrant rice plants.

In rice, BADH is encoded by two homologous genes called *BADH1* and *BADH2*. According to [46], the *BADH1* showed strong correlations with salt tolerance, while *BADH2* solely contributed to aroma synthesis in aromatic rice. A comparison of the two alleles of *BADH2* discovered that the deletion of eight bp in exon 7 and three SNP in exon 13 caused significant variations in aroma synthesis in aromatic rice [47]. Therefore, the responses of 2-AP contents under foliar sprays of MoS_2_FL to Cd stressed fragrant rice plants could be partly explained by the changes in the *BADH2* expression, which is a crucial gene in regulating 2-AP synthesis in scented rice [44,48]. Our results showed that there existed a negative relationship between 2-AP content and *BADH2* expression, which also agrees with previous findings [5]. According to [49], down-regulating the expressions of *OsBADH2* gene substantially enhanced the 2-AP contents. Ref. [50] showed negative relations between 2-AP and *BADH2* expression in fragrant rice cultivars in comparison to non-scented cultivars. Ref. [31] reported that the significant increase in 2-AP content under different temperature regimes was caused by the down-regulation of the *BADH2* recessive allele in aromatic rice. Similarly, in the current experiment, the reduced transcriptional levels of *BADH2* gene under foliar sprayed MoS_2_FL treatments might have induced lower BADH activity and thus accounted for higher 2-AP content.

Taken together, the present study suggests that exogenous application of MoS_2_FL was associated with improved 2-AP accumulation in fragrant rice cultivars, along with coordinated changes in precursor levels, enzyme activities, and transcript abundances of genes related to the 2-AP biosynthesis pathway. Despite these promising effects, a key limitation of this study is the absence of a conventional molybdenum fertilizer or bulk Mo treatment. Therefore, it remains unclear whether the observed responses are driven by the nutritional role of molybdenum, the nanoscale properties of MoS_2_FL, or a combination of both. Future studies incorporating bulk Mo sources and conventional molybdenum fertilizers will be valuable for distinguishing the specific contribution of nanomaterial properties to the observed physiological and metabolic responses.

## 5. Conclusions

This study provides evidence that MoS_2_FL application is associated with enhanced antioxidant defense and improved aroma formation in fragrant rice under cadmium stress, possibly through coordinated changes in the 2-AP biosynthesis pathway. Cadmium toxicity severely impaired antioxidant capacity and disrupted aroma-related metabolic processes, whereas MoS_2_FL application alleviated oxidative damage, improved antioxidant enzyme activities, and promoted 2-AP accumulation. These findings suggest that nano-enabled modulation of antioxidant and metabolic responses may contribute to improved aroma quality and stress tolerance in fragrant rice. Overall, this study highlights the promising potential of MoS_2_ nanoflakes as a sustainable nanotechnology-based approach for enhancing crop quality and abiotic stress resilience in heavy metal-contaminated agricultural systems.

## Figures and Tables

**Table 1:** Primer sequences of target genes used for qRT-PCR analysis.

Genes	Strands	5′⟶3′ Primer Sequence	(Tm °C)	Accession Number
*P5CS2*	F	GAGGTTGGCATAAGCACAG	57	AP014957.1
	R	CTCCCTTGTCGCCGTTC		
*PRODH*	F	TCATCAGACGAGCAGAGGAGAACAGG	56	AP014966.1
	R	CCCAGCATTGCAGCCTTGAACC		
*BADH2*	F	GGTTGGTCTTCCTTCAGGTGTGC	57	AB09683
	R	CATCAACATCATCAAACACCACTAT		
*DAO*	F	TCGTTCGCATCAAGGTTGG	55.5	AP014960.1
	R	TCAGACAGAAGGGTGCCGTA		
*ACTIN*	F	TGCCAAGGCTGAGTACGACGA	57	Os03g50885
	R	CAAGCAGGAGGACGGCGATA		

## Data Availability

Data is contained within the article and Supplementary Materials.

## Supplementary Materials

The following supporting information can be downloaded at: https://www.mdpi.com/article/10.3390/antiox15070817/s1, Figure S1. Effects of foliar application of molybdenum disulfide nanoflakes (MoS_2_FL) on cadmium (Cd) concentration in roots (A), shoots (B), leaves (C), and grains (D) of fragrant rice cultivars Meixiangzhan-2 and Basmati under Cd stress (50 mg kg^−1^ soil). Values represent means ± SE of four biological replicates (n = 4). Different lowercase letters indicate significant differences among treatments according to Tukey’s honestly significant difference (HSD) test at *p* < 0.05.
