# Supplementary material for: Nano-Molybdenum Disulfide Enhances Antioxidant Defense and Aroma Formation in Fragrant Rice Under Cadmium Stress via Modulation of 2-Acetyl-1-Pyrroline Biosynthesis
Source: Antioxidants (Basel). 2026 Jun 29;15(7):817. doi: 10.3390/antiox15070817 (PMC13404246; doi:10.3390/antiox15070817)
Supplement: Supplementary file 1 [file antioxidants-15-00817-s001.zip › antioxidants-4351178-supplementary.pdf]

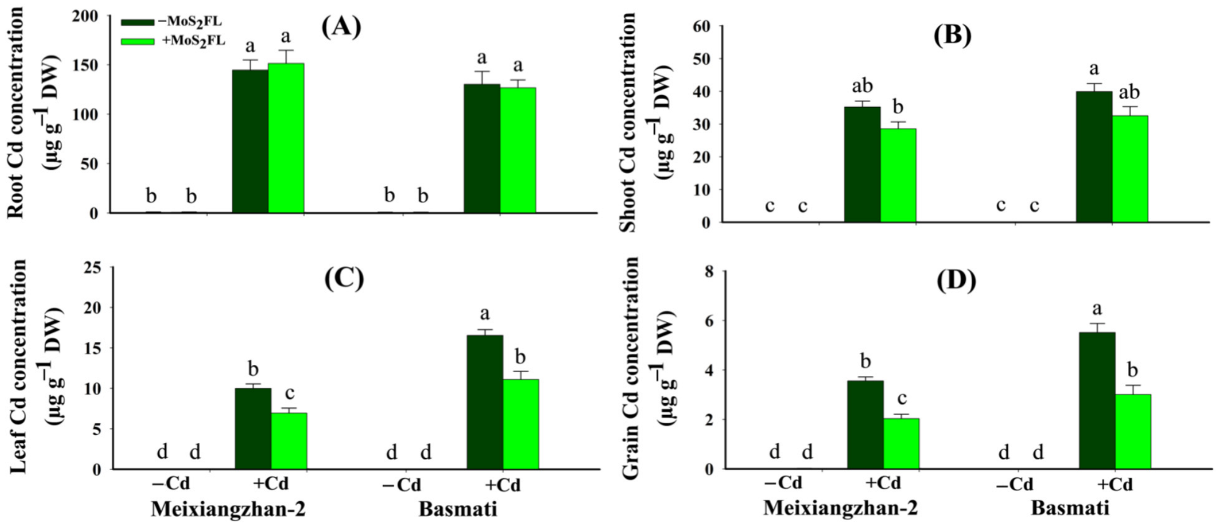

Supplementary Figure S1. Effects of foliar application of molybdenum disulfide nanoflakes (MoS_2FL) on cadmium (Cd) concentration in roots (A), shoots (B), leaves (C), and grains (D) of fragrant rice cultivars Meixiangzhan-2 and Basmati under Cd stress (50 mg kg^{-1} soil). Values represent means ± SE of four biological replicates (n = 4). Different lowercase letters indicate significant differences among treatments according to Tukey's honestly significant difference (HSD) test at $P < 0.05$.
